# Interseeding cover crops into established corn fields affects invertebrate abundance and diversity and elevates the activity of generalist predators

**DOI:** 10.1093/jisesa/ieag096

**Published:** 2026-09-16

**Authors:** Michael M Bredeson, Jonathan G Lundgren

**Affiliations:** Department of Natural Resource Management, South Dakota State University, Brookings, SD, USA; Blue Dasher Farm, Ecdysis Foundation, Estelline, SD, USA; Blue Dasher Farm, Ecdysis Foundation, Estelline, SD, USA

**Keywords:** biocontrol, intercropping, covers, interseeding, regenerative

## Abstract

Ecologically simplified corn fields lack the plant diversity-derived resources necessary for supporting robust beneficial insect populations capable of preventing pest outbreaks. Interseeding cover crops into established corn is a way that farmers can diversify cropland plant communities and restore ecosystem functions. Here, we examine how foliar, epigeic, and subterranean arthropod communities differ between corn monocultures and corn interseeded with cover crops. We also examine how predator activity and predation are affected by interseeded cover crops in corn fields. Predators, herbivores, numerous individual taxa, and all combined arthropods were more abundant on the soil surface in interseeded corn fields. Adding cover crops also increased the number of epigeic species. Within the subterranean environment, community structure was similar in the bare soil and interseeded corn fields, and only 4 commonly collected species differed in abundance. Below-ground arthropod diversity increased when cover crops were interseeded in corn fields. Community characteristics in the corn foliage were not affected by the cover crops. Twice as many wax moth (*Galleria mellonella (L.)*) sentinel larvae were eaten by generalist predators in cover-cropped corn (45.5%) than in corn monocultures (22.6%). The effect of cover crops on corn stand density and yield varied significantly between study locations. Diversification of the corn agroecosystem by interseeding cover crops had a spatially compartmentalized effect on invertebrates in corn fields, favoring the abundance and diversity of surface-dwelling invertebrate fauna and predator activity. This management tool should be further explored by researchers and farmers, as it is a viable method for conserving biodiversity and bolstering biological control of crop pests.

## Introduction

In 2020, 37.2 million hectares of corn were planted in the United States (USDA-NASS 2026), 4.9% of the entire landmass of the 48 contiguous states (USDA-ERS 2026). In especially agrarian states (e.g. Iowa), the percentage of land occupied by this 1 plant species is much higher (38.9%), and this dominance affects regional food webs. Corn monocultures have replaced what was historically perennial grasslands (Rashford et al. 2011, Wimberly et al. 2017), plant-diverse habitats that support greater faunal biodiversity (Schmid et al. 2015, Bredeson et al. 2026, Pecenka et al. 2026). Diverse habitats also perform numerous ecosystem functions that are lost or impaired within agroecosystems (DeFries et al. 2004, Fiedler et al. 2008). One such function is the maintenance of herbivore communities at sub-outbreak population sizes (Landis et al. 2005, Moreira et al. 2016). When a habitat has a diverse plant community, herbivore population growth can be curtailed through a multitude of top-down and bottom-up antagonisms (Landis et al. 2005, Moreira et al. 2016). In diversified agricultural landscapes, herbivore outbreaks are kept in check in through biological control and other processes, and fewer than 5% of insects in healthy agroecosystems are pests (Bredeson et al. 2026, Lundgren et al. 2026, Pecenka et al. 2026). In simplified crop fields, where these natural process are disabled, farmers often resort to chemical control of herbivores (Fausti et al. 2012, 2018). Adding plant diversity to would-be monocultures can positively affect ecosystem functions including crop pest control (Gurr et al. 2017, Bennion et al. 2020; Jaworski et al. 2023). It should be noted that under some circumstances, diverse agroecosystems may generate neutral community responses, increase herbivore abundance, and reduce agronomic productivity (Bröcher et al. 2023).

Despite the widespread use of corn monocultures throughout industrialized agriculture, corn was historically grown with other plant species, a practice still commonly used in subsistence agriculture (Landon 2008). Advancements in agricultural technology and research that reveal plant synergisms have led to a recent increase in farmland being planted with multiple plant species simultaneously (CTIC 2017). Some corn producers are accomplishing this in their fields by planting cover crops (non-crop plants) between established rows of corn (Lundgren et al. 2026). Cover crop species can be selectively chosen to add benefits to the ecosystem without interfering with crop growth or harvest (Wallander et al. 2022).

By providing alternative foods and microhabitats, interseeding corn with cover crops has the potential to alter agroecosystem habitats in ways that make these production areas more suitable for a diversity of arthropod species, not just those whose life histories are specifically adapted to corn (Huss et al. 2022, Mir et al. 2022). Nectar and pollen produced by cover crops and alternative prey species that consume cover crop tissues are all resources by which natural enemies can sustain themselves in the absence of crop pests (Lundgren 2009, Manandhar and Wright 2016). Adding plant diversity during the corn growing season by incorporating cover crop mixtures may provide a means by which farmers can mimic the diversity and function of natural systems.

We explored how interseeding cover crops affects invertebrate community structure and predation in corn production systems of eastern South Dakota. The main null hypotheses we tested were (i) interseeded cover crops have no effect on insect community structure and the abundances of herbivores, predators, and detritivores, (ii) interseeded cover crops in corn do not affect sentinel predation rates on the soil surface, (iii) invertebrate communities in the foliage, on the soil surface, and within the soil respond similarly to treatment across the season, and (iv) interseeded cover crops have no effect on corn yield or density. The results of this study hold important implications as conservation of biodiversity on farmland becomes an ever more pressing issue.

## Methods

### Field Sites

Research was conducted at 3 sites over 2 years. Farmers applied intercropping according to their operations’ circumstances, leading to slightly different study designs that better captured real-world farming scenarios. At each site, 8 plots measuring 42 × 42 m each were established in a 2 × 4 orientation and were separated by 15 m of corn.

At site 1, corn (Elk Mound Seed Company, Elk Mound, Wisconsin, 54739; variety: EMS 8100; maturity: 80 d) was no-till planted on 26 May 2017 at 79,000 seeds/ha in rows 76-cm apart. Roundup (rate: 2.34 liter/ha; a.i.: glyphosate; Monsanto, St. Louis, Missouri, 63167) was applied as a pre-plant herbicide. No post-emergent herbicides or fertilizers were used at site 1.

At site 2, corn (Legend Seeds, Inc., De Smet, SD, 57321; variety: A10946, A10258; maturity: 97 d) was planted on 5 May 2017 at the same density and row spacing as in site 1. The field was cultivated prior to planting for seedbed preparation. SureStart II (rate: 2,923 ml/ha; active ingredients: acetochlor, flumetsulam, and clopyralid; Dow AgroSciences, Indianapolis, Indiana, 46268) was applied on 1 May as a pre-plant herbicide. Fertilizer was broadcasted into research fields at a rate of 157 kg nitrogen/ha as urea, 67 kg phosphorous/ha as MicroEssentialsSZ (Mosaic, Plymouth, Minnesota, 55441) and 56 kg potassium/ha as potash. Insecticides were not used at either site 1 or site 2.

At site 3, all plots were cultivated prior to planting. Corn (Blue River Organic Seed; Ames, Iowa, 50014; variety: P1000684; maturity: 96 d) was planted on 26 May 2018 in 76 cm-spaced rows at a population of 79,000 seeds/ha. Research plots were sized and oriented similarly to those in site 2. On 20 May, pre-emergent herbicide, SureStart II (rate: 2923 ml/ha), was applied in plots with no further herbicide use during the remainder of the growing season. Fertilizer was broadcasted into plots at a rate of 157 kg/ha nitrogen as urea, 56 kg/ha potassium as potash, and 56 kg/ha phosphorous as diammonium phosphate. No insecticides were used during 2018.

An 8-species cover crop mixture (coated in calcium carbonate [1:1, seed: CaCO_3_, by weight]) of hairy vetch (*Vicia villosa*, 3.5 kg/ha), lentils (*Lens culinaris*, 3.5 kg/ha), mung beans (*Vigna radiata*, 5 kg/ha), oats (*Avena sativa*, 5 kg/ha), flax (*Linum usitatissimum*, 9 kg/ha), cereal rye (*Secale cereale*, 14.6 kg/ha), and field peas (*Pisum sativum*, 14.6 kg/ha) was planted into 4 of the established plots in an alternating pattern. This mix was developed together with Green Cover Seed (Bladen, Nebraska) to represent common cover crop species in different functional and taxonomic guilds that would be well adapted for corn fields. At sites 1 and 2, this seed was broadcasted immediately following corn emergence (site 2: 16 May; site 1: 2 June). At site 3, seeds were planted following corn emergence using a 7-row, homemade, tractor-drawn cover crop interseeder (Supplementary Fig. S1). This device planted a single row of cover crops between each pair of corn rows. At site 1, germination and growth of the cover crop were extensive, and weed control was poor, which contributed to yield reductions in the crop. At sites 2 and 3, there was vigorous cover crop growth until the corn crop formed a canopy, at which point the cover crop laid down and grew laterally to cover the soil surface. At site 2, broadcasting the seed led to reduced stand density in the cover crops, with small topographic changes within the field leading to local pockets of relatively dense cover crop. The interseeder substantially improved ­uniformity in cover crop germination and subsequent plant density.

### Insect Sampling

We collected soil-dwelling arthropods from a total of 160 cores taken from each site per year and 480 cores across all 3 sites. Each plot was sampled 4 times in each year (corn stages: V2 [2 leaf; 9 June], V4 [4 leaf; 27 June], V8 [8 leaf; 16 July], and anthesis [crop pollination]; 2 August) by taking 5 soil cores (diameter: 11 cm, depth: 10 cm) within corn rows within each of the 8 plots using haphazard random sampling where the sampler took care not to bias location of samples. Soil invertebrates were extracted for 7 d using Berlese funnels into 70% ethanol. Arthropods were stored in 70% ethanol until identification and curation.

Epigeic invertebrates (those that dwell on the soil surface) were collected from 96 quadrats from each site over the course of a field season and 288 samples across all 3 sites. Four quadrat collections were taken from each plot on 3 dates (corn stages: V4, V8, and anthesis) per year at each site. Samples were selected randomly by tossing the quadrat onto the soil surface into an area of the field. Manual aspirators were used to collect arthropods off the soil’s surface within confined 0.5 × 0.5 m sheet metal quadrats pressed >2 cm into the soil to prevent arthropod escape. Before leaving a sampled plot, arthropods from each quadrat were placed in 70% ethanol until identified.

Foliar arthropods were collected using whole-plant dissections (Lundgren et al. 2015) from a total of 360 corn plants per site, per year, or 1,080 plants across all 3 sites. At each site, plots were sampled 3 times (corn stages: V4, V8, and anthesis). On each sample date, 15 corn plants were collected using ­haphazard random sampling where the sampler took care not to bias location of samples (>5 m from the plot edge) and plants were dissected on white cotton sheets. Invertebrates were ­identified upon sight to species level, or the lowest possible taxonomic unit, and recorded.

### Yield

Yield and plant density were measured following corn maturation. Corn ears were collected, and plants were counted within a 3 m row section at 4 points located 8, 16, 24, and 32 rows from the plot’s edge; row series were sampled so the final collection points represented a diagonal pattern across each field. Corn kernels were removed using a hand-sheller (item number: 530065; Premier1Supplies, Washington, Iowa, 52353) and weighed (weights were adjusted to 15% moisture for comparison, which is standard moisture content for on-farm corn storage).

### Predation

Predation experiments were conducted at sites 1 and 2 (we did not have the resources to examine predation at site 3). To measure general epigeic predatory activity, wax moth (*Galleria mellonella*) larvae (*n* = 40 per plot) were used as sentinel baits in both treatments. Although wax moth larvae are not crop pests, they have been used successfully in previous field experiments for comparing generalist predatory activity between different agricultural habitats (Lundgren et al. 2006, Meehan et al. 2012). Active, prepupal larvae were individually pinned (#0 black enameled insect pins; model: 01.10; Entochrysis, Pardubice, 53002, Czech Republic) to 1 cm tall pyramids made of sculpting clay (Sculpey original, Polyform Products Co. Inc., Elk Grove Village, Illinois, 60007) through the larvae’s posterior segments. Previous work has shown that predators are most abundant in corn fields around anthesis (Lundgren and Fausti 2015). Larvae were deployed in corn fields during anthesis within 1 h of being pinned to clay and were only used if obviously alive and were able to move. Preliminary work by our team showed that this 1-h interval was able to detect treatment effects on predation, without seeing all larvae consumed. Sentinel predation was always measured in the morning to avoid the warmest daylight hours. Clay pyramids were buried in the soil so that pinned larvae were presented flush with the soil surface. Once deployed, sentinels remained undisturbed for 1 h, at which time they were recollected to assess the proportion of sentinels that had been predated. Sentinels were considered eaten if there were invertebrates actively feeding or if the wax moth larva had been partially or wholly consumed. Predators present at sentinels were identified upon sight to species level, or the lowest taxonomic rank possible. Four rows received larvae (*n* = 10 each), spaced 3 m apart, and rows with sentinels were at least 4 m apart. In total, 640 wax moth sentinel larvae were deployed to assess predation. Methods for sentinel predation were modeled after those described by Lundgren and Fergen (2011).

### Data Analysis

The experimental unit is the field, with replicate fields across sites pooled into 2 treatments (site was not considered as a cofactor). The biological patterns in the data suggested that this was an appropriate approach. Shannon diversity index was calculated, and species richness reported for invertebrates in both treatments (interseeded and monoculture) and in each of the 3 habitat types (subterranean, epigeic, and foliar) at each site each year. Invertebrate species collected within soil and from the soil surface were categorized into 3 feeding guilds: predators, herbivores, and detritivores. Invertebrates collected on corn foliage were categorized into 2 feeding guilds: predators and herbivores. Mean ± SEM arthropods per m^2^ of soil or per plant in the various arthropod feeding guilds from corn monocultures and interseeded corn were determined. Individual taxa were not included in feeding guilds if their life histories were unknown. Two-way repeated measures ANOVAs (rm-ANOVAs) coupled with Tukey’s HSD all-pairwise comparisons were conducted to examine the effect of treatment (monoculture and interseeded corn), corn stage (V4, V8, and anthesis), or an interaction of both on diversity, species ­richness, and arthropod abundance in the different corn field ­habitats. If an interaction was revealed, subsequent one-way ANOVAs were conducted to determine treatment differences for individual corn stages. Individual taxa driving overall trends in abundance for each habitat were determined by performing rm-ANOVAs on taxa representing ≥0.5% of the total arthropod community abundance within the soil on the soil surface or on corn foliage. To gain a better understanding of treatment effects on the invertebrate community, springtails (Collembola) and mites (Acarina) were excluded when determining common subterranean and epigeic taxa due to their extraordinary abundances. Two-way ANOVAs were conducted on corn yield and density to determine if there was a significant interaction between year and treatment. If an interaction existed, sub­sequent one-way ANOVAs tested treatment differences at ­individual sites.

Mean ± SEM percent sentinel predation per plot was determined for each treatment at individual sites and across all site years. A two-way ANOVA was used to determine treatment and site effects on rates of sentinel predation and if an interaction existed between the 2. Subsequent one-way ANOVAs were used to examine the levels of significance between treatments at individual study locations. Linear regressions were conducted to examine if correlations existed between diversity (Shannon H) or epigeic predators/m^2^ (during anthesis) and predation rate at the sites. Data analyses were conducted using Statistix 10 software (Analytical Software, Tallahassee, Florida, United States).

## Results

### Invertebrate Communities

A grand total of 63,868 invertebrates were collected across all sites and habitats. The invertebrate community consisted of 516 species from 22 orders, including: Araneae, Acarina, Cephalostigmata, Coleoptera, Collembola, Diplura, Diptera, Ephemeroptera, Hemiptera, Hymenoptera, Lepidoptera, Neuroptera, Odonata, Opiliones, Orthoptera, Protura, Pseudoscorpiones, Psocoptera, Stylommatophora, Thysanura, and 2 unidentified orders, 1 each from classes Chilopoda and Diplopoda.

### Subterranean Invertebrates

Interseeded corn possessed a greater diversity (Shannon H) of subterranean invertebrates than corn monocultures (*F* = 4.17, df = 1, 95, *P* = 0.05). There was a total of 361 species collected across both treatments with no difference in terms of species richness between monoculture and interseeded fields (*F* = 2.00, df = 1, 95, *P* = 0.17; monoculture: 55.25 ± 5.32, interseeded 64.50 ± 6.39 species). Across treatments, both species richness and diversity varied between corn stages (*F* = 12.08, df = 3, 95, *P* < 0.01 and *F* = 4.17, df = 3, 95, *P* < 0.01, respectively), with corn having the greatest number of species and diversity during anthesis compared to all earlier sampled stages.

**Fig. 1.**
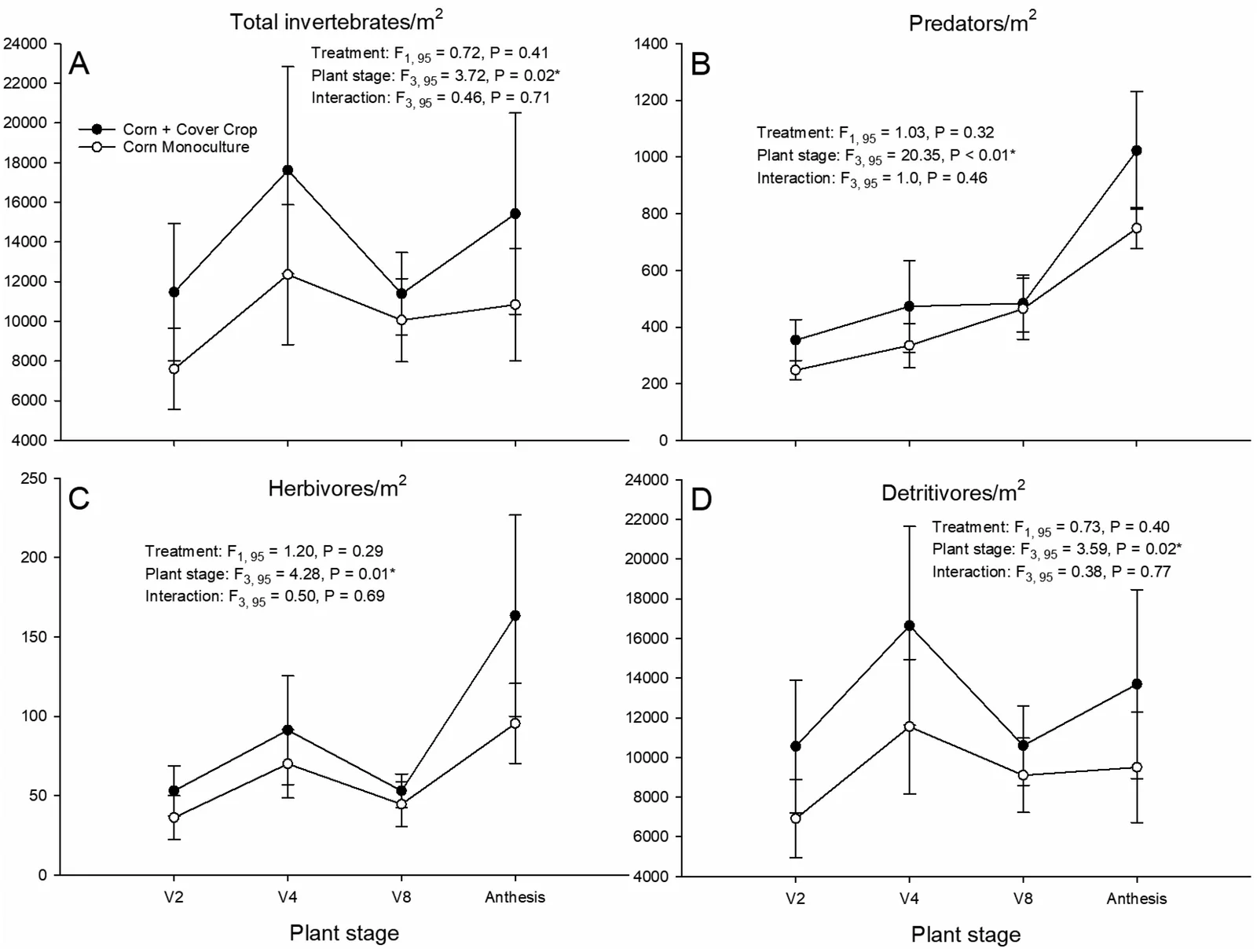
Total invertebrates (A), predators (B), herbivores (C), and detritivores (D) in the soil column of corn monocultures (open circles) and corn interseeded with cover crops (dark circles). Data presented are mean ± SEM arthropods collected in the top 10 cm of the soil column from 4 fields at 3 locations (*n* = 12). Samples were taken during 4 corn stages throughout the growing season (V2, V4, V8, and anthesis). Results of rm-ANOVAs are also presented (α = 0.05) with significant differences between treatments denoted with an asterisk.

A total of 45,797 invertebrates were extracted from soil cores throughout the duration of the experiment, with 19,254 from corn monocultures and 26,543 from corn interseeded with cover crops. Despite collecting 7,289 (38%) more invertebrates from interseeded plots, total arthropods per square meter were not significantly different between treatments (*F* = 0.72, df = 1, 95, *P* = 0.41, Fig. 1A). When the community was separated into feeding guilds (predators: *n* = 167 species; herbivores: *n* = 38 species; detritivores: *n* = 41 species), an interseeded cover crop did not affect invertebrate abundance for predators (*F* = 1.03, df = 1, 95, *P* = 0.32), herbivores (*F* = 1.20, df = 1, 95, *P* = 0.29), or detritivores (*F* = 0.73, df = 1, 95, *P* = 0.40) (Fig. 1B–D).

Acarina and Collembola dominated the subterranean community, representing 56.83% and 33.01% of all individuals collected, respectively. Diplurans were the next most abundant group, representing 0.68% of collected specimens. Diplurans were much more abundant than the remaining taxa. Twenty-seven species each represented ≥0.5% of all collected subterranean arthropods, and although an overall treatment effect did not exist for the abundances of subterranean invertebrates, the abundances of 4 individual taxa differed between interseeded and monoculture corn. One Lycosidae spider species, a detritivorous Cryptophagidae beetle, and all Thripidae (herbivorous) had increased abundance when cover crops were present, whereas Beetle Larvae 15 (see Table 1 footnote for specimen description) was found less frequently in cover-cropped fields (Table 1).

### Epigeic Invertebrates

There were 298 epigeic invertebrate species collected over the season (in both treatments), with greater species richness in interseeded corn fields than in monocultures (*F* = 13.07, df = 1, 71, *P* < 0.01; monoculture: 39.50 ± 3.04 species, interseeded: 61.25 ± 4.03 species). Across treatments, invertebrate richness increased incrementally as the growing season progressed (*F* = 30.70, df = 2, 71, *P* < 0.01). Interseeding cover crops did not affect the diversity (Shannon H) of surface-dwelling arthropods (*F* = 0.00, df = 1, 71, *P* = 0.99), but invertebrate diversity varied among corn stages (*F* = 14.16, df = 2, 71, *P* < 0.01), with V4 corn being less diverse than corn at the V8 and anthesis stages.

**Fig. 2.**
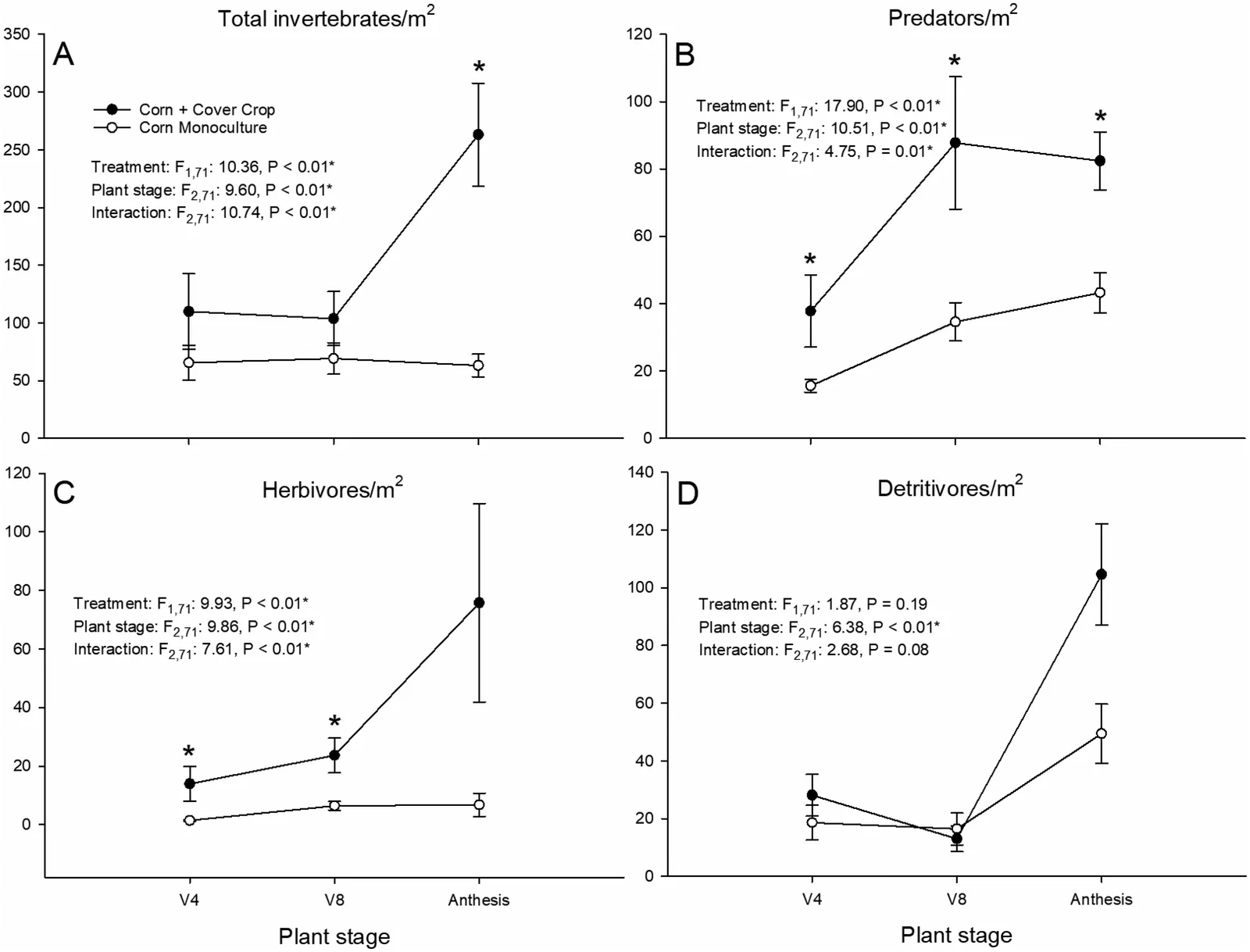
Total invertebrates (A), predators (B), herbivores (C), and detritivores (D) captured on the soil surface from corn monocultures (open circles) and corn interseeded with cover crops (dark circles). Data presented are mean ± SEM, generated from 4 fields at 3 locations (*n* = 12). Samples were collected per m^2^ at 3 plant stages throughout the growing season (V4, V8, and anthesis). Results of rm-ANOVAs are presented (α = 0.05) with significant differences between treatments denoted with an asterisk. Note the differences in scale on the *y* axis among the different trophic groups.

A total of 8,098 invertebrates were collected from the soil’s surface. Across all site years and sampling dates, more than twice as many invertebrates were collected from the epigeic environment in interseeded corn (*n* = 5,722) compared to corn monocultures (*n* = 2,376) (*F* = 10.36, df = 1, 71, *P* < 0.01; Fig. 2A).

The 5 most abundantly collected taxa from the soil surface were Collembola (all springtails, 24.46%), *Rhopalosiphum padi* (Aphididae) (12.07%), *Stenolophus comma* (Carabidae) (7.26%), Acarina (all mites, 6.74%), and *Elaphropus* sp. (Carabidae) (3.52%). Based on known life histories, 165 species were designated as predators, 52 species were classified as herbivores, and 16 were detritivores. Throughout the growing season, both predators and herbivores were more abundant in interseeded plots compared to monocultures (predators: *F* = 17.90, df = 1, 71, *P* < 0.01, herbivores: *F* = 9.93, df = 1, 71, *P* < 0.01; Fig. 2B and C), but detritivore abundance was statistically similar between treatments (*F* = 1.87, df = 1, 71, *P* = 0.19; Fig. 2D). Increased predator abundance on the soil surface in interseeded plots was largely driven by 3 predatory groups and 4 commonly collected individual taxa. Ground beetle (Carabidae: *F* = 5.42, df = 1, 71, *P* = 0.03), rove beetle (Staphylinidae: *F* = 4.53, df = 1, 71, *P* = 0.04), and spider (Araneae: *F* = 6.90, df = 1, 71, *P* = 0.02) abundances were higher in cover-cropped corn. Individually, predatory *Bembidion* sp. (*F* = 8.89, df = 1, 71, *P* = 0.01), *Nabis americoferus* (*F* = 5.50, df = 1, 71, *P* < 0.01), a Tetragnathid spider species (*F*_1, 71_ = 4.57, df = 1, 71, *P* = 0.04), and larval Coccinellidae (*F* = 16.69, *df* = 1, 71, *P* < 0.01) were more abundant when cover crops were present. For herbivores, significantly more bird cherry oat aphids (*R. padi: F* = 4.43, df = 1, 71, *P* = 0.05) and plant bugs (Miridae: *F* = 6.10, df = 1, 71, *P* = 0.02; note that some mirids are omnivorous, despite them being classified as herbivores) were found in the interseeded fields compared with the monoculture corn fields (Table 1).

Twenty-seven species individually represented ≥0.5% of all surface-dwelling arthropods (excluding the abundances of mites and Collembola). Of these 27, the abundance of 6 species, 2 herbivorous (*R. padi* and *Lygus* sp.) and 4 predatory (Tetragnathidae sp., Coccinellidae immatures, *N. americoferus*, and *Bembidion* sp.) were significantly increased in interseeded corn (Table 1). There were no species whose abundance was lower in interseeded corn compared to corn in monoculture.

An interaction existed between treatment and corn stage for surface-dwelling invertebrate abundance and for the abundance of predatory and herbivorous guilds (Fig. 2A–D). Total arthropod abundances did not differ during V4 and V8 plant stages, but during anthesis, more invertebrates were collected in interseeded plots (*F* = 19.23, df = 1, 23, *P* < 0.01). Predators were more abundant in interseeded plots during all of the sampled corn stages (V4: *F* = 14.06, df = 1, 23, *P* < 0.01; V8: *F* = 6.75, df = 1, 23, *P* = 0.02; anthesis: *F* = 4.15, df = 1, 23, *P* = 0.05), whereas herbivores were more numerous in interseeded plots during V4 and V8 (*F* = 7.89, df = 1, 23, *P* = 0.01 and *F* = 4.42, df = 1, 23, *P* = 0.05, respectively), but not anthesis (*F* = 0.19, df = 1, 23, *P* = 0.68).

### Foliar Invertebrates

In both interseeded and monoculture fields, there was a total of 66 species collected from corn foliage. Species richness did not differ between treatments (*F* = 0.13, df = 1, 71, *P* = 0.73; monoculture: 17.00 ± 1.24, interseeded 18.50 ± 1.40 species), but differed among corn stages (*F* = 36.58, df = 2, 71, *P* < 0.01), with greater richness measured at V8 and anthesis than at V4. Similarly, foliar arthropod diversity did not differ between corn monocultures and interseeded corn (*F* = 0.07, df = 1, 71, *P* = 0.79), but diversity differed among plant stages (*F* = 35.55, df = 2, 71, *P* < 0.01) with arthropods being more diverse during V8 and anthesis compared to V4 corn.

**Fig. 3.**
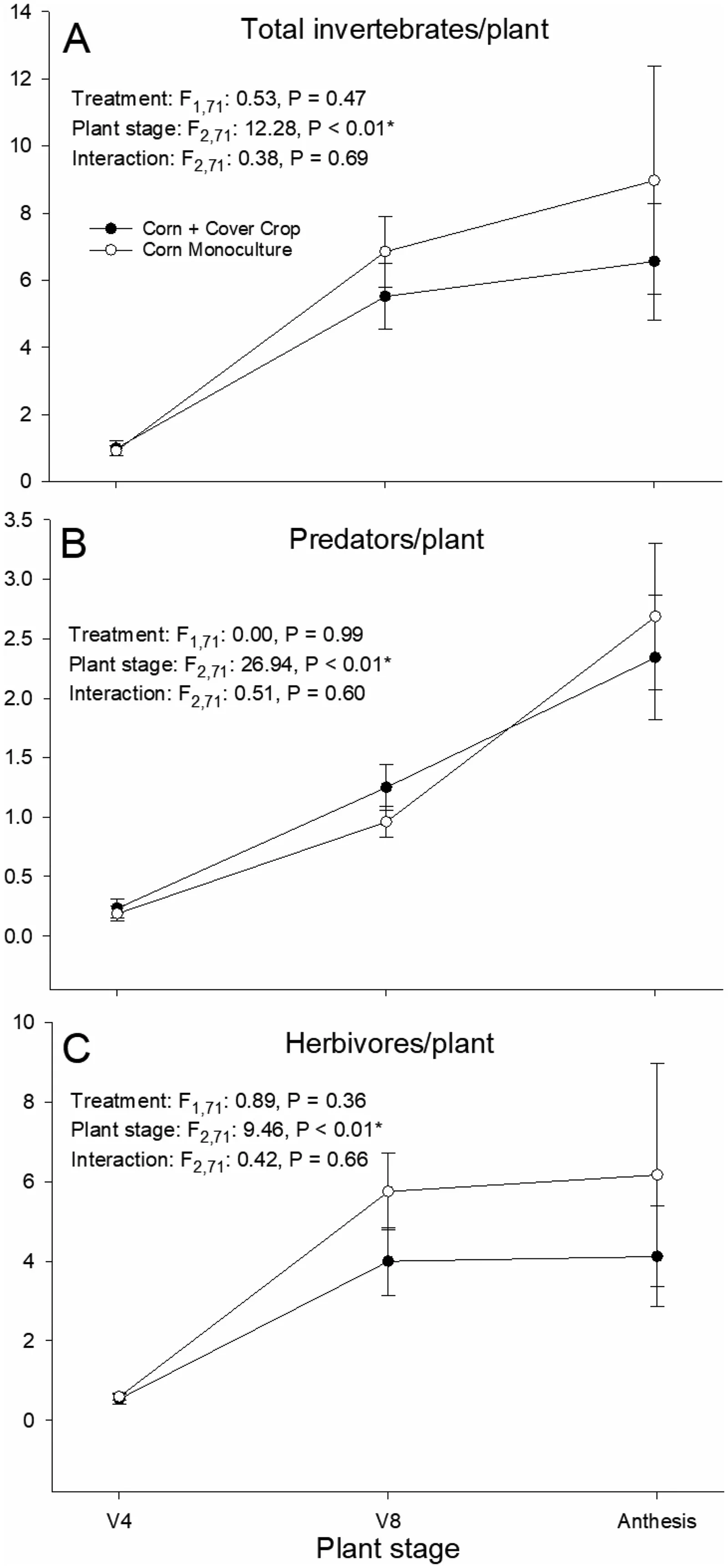
Total invertebrates (A), predators (B), and herbivores (C) collected from corn foliage at 3 plant stages from corn monocultures (open circles) and corn interseeded with cover crops (dark circles). Data presented are mean ± SEM arthropods collected per corn plant from 4 fields at 3 locations (*n* = 12) Results of rm-ANOVAs are also presented (α = 0.05) with significant findings denoted with an asterisk.

A total of 9,973 invertebrates were collected from corn foliage, with 4,681 from interseeded corn and 5,292 from corn monocultures. The abundance of total invertebrates per dissected corn plant did not vary between treatments (*F* = 0.53, df = 1, 71, *P* = 0.47, Fig. 3A). Likewise, interseeding corn with cover crops did not affect the number of predators (*n* = 29 species) or herbivores (*n* = 18 species) (*F* < 0.01, df = 1, 71, *P* = 0.99; *F* = 0.89, df = 1, 71, *P* = 0.36; *F* = 1.87, df = 1, 71, *P* = 0.19, respectively, Fig. 3B and C). Across treatments, abundance varied among corn stages for total arthropods, predators, and herbivores, with corn plants during anthesis possessing more invertebrates than V4 or V8 plants (Fig. 3).

Only 6 individual or grouped taxa represented ≥0.5% of total invertebrate abundance in corn foliage. Herbivorous thrips (Thripidae), corn leaf aphids (*Rhopalosiphum maidis* Hemiptera: Aphididae), and mites (Acarina) represented 34.44%, 32.61, and 1.81% of the total foliar community abundance, respectively. Minute pirate bugs (*Orius insidiosus*), spiders (all Araneae), and ladybeetle larvae represented 5.17%, 3.15%, and 0.53% of the total foliar predator community, respectively. None of these taxa significantly differed in population size between interseeded and monoculture corn plots.

Corn earworms (*Helicoverpa zea*), European corn borers (*Ostrinia nubilalis*), and northern corn rootworms (*Diabrotica barberi*) are dominant pests of corn (SDSU 2019), but in this study, only 10 individuals of each species were collected from a total 1,080 plants. Seven *H. zea* were collected from interseeded plots and 3 from monoculture, 2 *O. nubilalis* from interseeded and 7 from monoculture, and 8 *D. barberi* from interseeded and 2 from monoculture plots.

### Predation Experiments

Across all site years, 33.13% ± 4.37% of sentinel larvae were eaten in interseeded corn compared to only 19.69% ± 5.29% in corn monocultures, leading to a treatment effect that was very near the 0.05 probability significance level (*F* = 3.83, df = 1, 13, *P* = 0.07; Table 2). Variability between treatments at different study locations resulted in a significant interaction effect between treatment and site (*F* = 18.2, df = 3, 31, *P* < 0.01). At site 2, sentinels were consumed at a significantly higher rate in interseeded corn than in corn monocultures (site 2: *F* = 7.01, df = 1, 7, *P* = 0.04), whereas at site 1, sentinel predation was not different between treatments (site 1: *F* = 0.37, df = 1, 7, *P* = 0.57) (Table 2).

Of the 369 total predation events, we observed active feeding by 547 invertebrates on 211 sentinels at the conclusion of the 1-h deployment period. Crickets (Orthoptera: Gryllidae) were most commonly observed consuming sentinels (*n* = 330, 60.3%), followed by ants (Hymenoptera: Formicidae; *n* = 119, 21.8%), harvestmen (Opiliones: Phalangiidae; *n* = 37, 6.8%), and ground beetles (Carabidae; *n* = 31, 5.7%) (remaining grouped taxa presented in Table 3). When comparing sentinel predation rates among both treatments at sites 1 and 2 to epigeic predator abundance and species diversity (Shannon H) during anthesis, no significant correlations were revealed (­predator abundance: *F* = 1.81, df = 1, 16, *P* = 0.20, community diversity: *F* = 0.50, df = 1, 16, *P* = 0.49).

### Corn Yield and Density

Significant interactions between treatment and site year existed for both corn yield (*F* = 44.26, df = 2, 23, *P* < 0.01) and corn density (*F* = 14.47, df = 2, 23, *P* < 0.01), owing to the uniquely large treatment effect observed in both measurement types at site 1. At site 1, corn plant density was greater in interseeded plots (*F* = 58.93, df = 1, 7, *P* < 0.01), but corn yield was reduced in the presence of cover crops (*F* = 117.97, df = 1, 7, *P* < 0.01). At site 2, corn density was also unchanged between treatments, but yield was marginally greater in cover-cropped corn (*F* = 4.74, df = 1, 7, *P* = 0.07). Neither yield nor density differed between interseeded and monoculture corn plots at site 3 (Table 4).

## Discussion

Over 63,000 specimens collected across several plant stages and from multiple corn field strata resulted in a robust bioinventory of invertebrates occurring in corn fields and revealed spatially focused effects of intercropping on invertebrates and their function. The demographics of this community were relatively consistent with other corn field bioinventories described previously (Stevenson et al. 2002, Lundgren et al. 2015, Welch and Lundgren 2016, LaCanne 2017, Kirk et al. 2025, Bredeson et al. 2026). Corn fields hosted thousands of invertebrates per m^2^, representing hundreds of species from a total of 22 orders. By far, most invertebrates (approximately 70% of specimens and 70% of species) were collected in the top 10 cm of the soil column. These numbers are particularly stark when one considers the relative spatial area sampled over the study; soil communities were assessed over a total area of 15 m^2^, whereas the epigeal and foliar communities were collected from 72 and 137 m^2^, respectively. Collembola and mites dominated the soil community. Soil invertebrates represent a significant source of nutrients and ecosystem functions within the subterranean environment; this is important to note considering the soil invertebrate community can be influenced by farm management (Altieri 1999, Landis et al. 2000, Pearsons and Tooker 2017). Cropland arthropod communities provide an abundance of services to farmers, but their diversity is lower than invertebrate communities from prairie habitats, ecosystems replaced by grain production (Standen 2000, Nemec et al. 2014, Schmid et al. 2015, Wimberly et al. 2017, Bredeson et al. 2026). Within simplified corn monocultures, invertebrate pests receive special attention from land managers. Despite an absence of insecticide use in fields assessed for community characteristics, corn pests were never found at economically actionable levels, and foliar (corn earworms and European corn borers) and root pests (corn rootworms) of special concern were particularly scarce (fewer than 1 per 100 dissected corn plants for foliar pests, and corn rootworm larvae were not found in soil cores). These results call into question the need for prophylactic insecticidal products meant to control these arthropods, which has been pointed out by previous researchers (Hutchison et al. 2010, Bredeson and Lundgren 2015, Mourtzinis et al. 2019, Lundgren et al. 2026, Pecenka et al. 2026). Clearly, a broader view of biodiversity that transcends managing the handful of problematic species to managing entire insect communities and their function seems justified by the current study (Coll and Wajnberg 2017).

The 3 invertebrate communities (foliar, epigeal, and soil) responded differently to interseeding cover crops over the season. The development of crop and intercrop over the season represents major shifts in habitat structure, microclimate, and resource availability. In turn, these changes affect the trophic interactions within agroecosystems. There was a significant steady increase in the number of foliar predators found within the corn field as the plants aged, whereas the number of herbivores plateaued after the V8 plant stage (Fig. 3). This pattern differed in epigeal and soil invertebrates, which experienced increasing herbivore and predator abundance later in the season (Figs 1 and 2). Although sampling dates significantly affected the dominant functional guild in the soil (detritivores), clear seasonal patterns in their abundance were not evident (there was a seasonal peak in abundance at V4). These seasonal patterns in insect abundance become important from the perspective of pest management, and interseeding cover crops affected the seasonal distributions of invertebrates, especially on the soil surface. We hypothesize that there is a widespread restructuring of the food web that helps to suppress pest outbreaks, and predation is only 1 mechanism whereby this occurs.

Interseeding covers into standing corn increased the diversity and abundance of the invertebrate community over corn planted in bare soil, a pattern commonly observed in other studies and systems (Slosser et al. 2000, Lundgren 2009, Alarcón-Segura et al. 2022, Huber et al. 2022, Pierre et al. 2022, Jarvinen et al. 2023, Bredeson et al. 2026). Foliar communities were largely unaffected by the cover crop. One reason for this may have been that the low-growing cover crops attracted invertebrates that are uniquely adapted to this habitat stratum (Horton et al. 2009). This pattern has been seen in orchard systems as well, where an enhanced ground-level plant community improved habitat for arthropods, but those animals were not observed moving into the orchard canopy (Altieri and Schmidt 1986, Horton et al. 2009, Fenster et al. 2021). Within the soil column, invertebrate abundances were consistent between interseeded corn and monocultures. We imagine that the rate of dispersal of invertebrates throughout this habitat is curtailed relative to dispersal rates on the soil surface and in foliar habitats (Ojala and Huhta 2001). It is feasible that communities within annual, ephemeral cropping systems simply do not have the time to respond to added plant diversity within the first season of implementing interseeded cover crops (Wiedenmann and Smith 1997). The observation that species diversity (Shannon H) increased belowground in interseeded corn fields may be an initial rebalancing of the successional community in response to the cover-crop mediated habitat change (Longcore 2003). We hypothesize that most niches in the soil column in this disturbed habitat were occupied by species that are adapted to monoculture cropland (Samu and Szinetar 2002). As plant diversity in the habitat increased, it began to change the relative abundances of the species that were there. If this is true, then we might expect richness and diversity to increase in the interseeded cropland over monocultures over time (Siemann et al. 1999, Suding and Gross 2006).

The epigeic community was most strongly influenced by the addition of interseeded cover crops. More than twice as many specimens were collected on the soil surface of interseeded fields, and all functional guilds (except for detritivores) had increased abundance significantly relative to in monocultures. Conditions on the soil surface within vegetationally diverse fields also resulted in a greater number of species inhabiting this environment. We expected that this community would be most affected due to their proximity to resources made available by cover crops (as seen in Horton et al. 2009). Diversifying plant assemblages directly affects invertebrate communities by ameliorating harsh abiotic conditions (Orr et al. 1997), increasing habitat structural complexity and niche partitioning (Langellotto and Denno 2004, Letourneau et al. 2011) and providing a variety of nutritional sources (Venzon et al. 2006, Lundgren 2009). Exactly how diversification of plant communities alters habitat suitability for invertebrates is often categorized into these simplified cause-and-effect relationships. In truth, multi-trophic ecological interactions within even the most simplified agroecosystem become infinitely complex (Lundgren and Fausti 2015), and in some ways, it is impossible to predict why invertebrate communities respond the way that they do to habitat manipulations except for in broad patterns.

Interseeding cover crops increased predation on the soil surface in corn fields. Adding plant diversity into agricultural production systems often elevates biological control (Altieri and Schmidt 1986, Lundgren and Fergen 2010, Bickerton and Hamilton 2012). One explanation is that community structure and function are linked; changes in predator community structure in response to plant diversification are responsible for increasing predation rates. This was not what we observed. Predator abundance and diversity were not correlated with consumption rates per field. Factors known to influence predation include relative prey and non-prey food sources in a habitat (Nomikou et al. 2010), intraguild interactions (Frank et al. 2010), structural complexity of the habitat that may impede or enhance foraging by predators (Finke and Denno 2002), etc. Within interseeded fields, it is likely that a combination of altered behavioral factors (e.g. foraging behavior), not predator community structure, interacted to increase predation by natural enemies.

The response of crop yield to undersown cover crops is highly variable depending on a host of factors such as nutrient availability, weed pressure, soil moisture, cover crop species, and sowing times (Abdin et al. 1998, Curran et al. 2018). It is possible that reduced yield in cover-cropped fields at site 1 was a result of competition between corn and cover crops for nutrients in fields not receiving fertilizer. Interseeded fields at this location also possessed a challenging weed population, further depleting available soil fertility. Alternatively, marginally increased corn yield in cover-cropped fields at site 2 reflects the results of studies identifying synergisms in mixed-cropping systems. For example, Rerkasem and Rerkasem (1988) recorded an increase in corn yield and leaf tissue nitrogen when ricebean (*Vigna umbellata*) was grown in close proximity. Meng et al. (2015) help explain this synergistic phenomenon by observing the transfer of legume-fixed nitrogen to corn via mycorrhizal fungi. Within published literature, the synergistic effects of intercropping typically leads to a yield advantage over monocultures (Yu et al. 2015). Crop establishment and density also benefit from companion plants by protecting young seedlings from adverse abiotic conditions such as damaging wind and erosion (Rinehart 2006) or biotic factors such as stand-reducing herbivores (Frank et al. 2010). These protective effects may have contributed to improved corn stand density in interseeded fields at site 1.

**Table 1.** Invertebrate abundance (mean ± SEM) in corn plots interseeded with cover crops and in corn monocultures summed across sampled corn stages in the corn foliage, on the soil surface, and in the soil column

Order	Family	Genus, species	Interseeded	Monoculture	Statistics
**Subterranean community**					
** Invertebrates**			55,905.85 ± 14,908.85	40,858.27 ± 9,681.05	*F*_1,95_ = 0.72, *P* = 0.41
** Predators**			2,334.27 ± 469.24	1,797.39 ± 245.26	*F*_1,95_ = 1.03, *P* = 0.32
** Herbivores**			360.75 ± 88.59	246.16 ± 55.75	*F*_1,95_ = 1.20, *P* = 0.29
** Detritivores**			51,491.95 ± 14,204.70	37,066.14 ± 9,169.67	*F*_1,95_ = 0.73, *P* = 0.40
** Araneae**	Lycosidae	sp. 1	118.84 ± 39.07	29.71 ± 13.24	*F*_1,95_ = 4.67, *P* = 0.04*
** Coleoptera**	Cryptophagidae	sp. 1	50.93 ± 15.36	14.85 ± 4.91	*F*_1,95_ = 5.01, *P* = 0.04*
** Coleoptera**		larva 15	6.37 ± 6.37	48.81 ± 19.69	*F*_1,95_ = 4.21, *P* = 0.05*
** Thysanoptera**	Thripidae	sp. 1	25.47 ± 10.86	4.24 ± 2.86	*F*_1,95_ = 5.77, *P* = 0.03*
**Epigeal community**					
** Invertebrates**			476.83 ± 80.49	198.00 ± 32.00	*F*_1,71_ = 10.36, *P* < 0.01*
** Predators**			207.91 ± 23.63	93.41 ± 13.19	*F*_1,71_ = 17.90, *P* < 0.01*
** Herbivores**			113.33 ± 31.01	14.58 ± 4.54	*F*_1,71_ = 9.93, *P* < 0.01*
** Detritivores**			145.67 ± 40.12	84.42 ± 19.82	*F*_1,71_ = 1.87, *P* = 0.19
** Araneae**			66.25 ± 13.22	28.00 ± 6.11	*F*_1,71_ = 6.90, *P* = 0.02*
** Coleoptera**	Carabidae		76.17 ± 19.19	29.17 ± 6.25	*F*_1,71_ = 5.42, *P* = 0.03*
** Hemiptera**	Miridae		22.92 ± 8.04	2.83 ± 1.22	*F*_1,71_ = 6.10, *P* = 0.03*
** Coleoptera**	Staphylinidae		5.17 ± 1.06	2.58 ± 0.60	*F*_1,71_ = 4.53, *P* = 0.05*
** Coleoptera**	Tetragnathidae	sp. 1	2.50 ± 0.57	1.08 ± 0.34	*F*_1,71_ = 4.57, *P* = 0.04*
** Hemiptera**	Nabidae	*Nabis americoferus*	6.42 ± 1.80	1.83 ± 0.77	*F*_1,71_ = 5.50, *P* = 0.03*
** Coleoptera**	Coccinellidae	Immatures	4.50 ± 0.88	0.75 ± 0.25	*F*_1,71_ = 16.69, *P* < 0.01*
** Coleoptera**	Carabidae	*Bembidion* sp.	14.33 ± 2.49	6.25 ± 1.03	*F*_1,71_ = 8.89, *P* = 0.01*
** Hemiptera**	Miridae	*Lygus* sp.	16.50 ± 5.53	1.08 ± 0.47	*F*_1,71_ = 7.72, *P* = 0.01*
** Hemiptera**	Aphididae	*Ropalosiphum padi*	75.00 ± 32.28	6.42 ± 4.35	*F*_1,71_ = 4.43, *P* = 0.05*
** Foliar community**					
** Invertebrates**			13.06 ± 2.71	16.73 ± 4.24	*F*_1,71_ = 0.53, *P* = 0.47
** Predators**			3.83 ± 0.64	3.84 ± 0.74	*F*_1,71_ = 0.00, *P* = 0.99
** Herbivores**			8.65 ± 2.05	12.51 ± 3.54	*F*_1,71_ = 0.89, *P* = 0.36

Data are presented this way to give relative abundances of invertebrate taxa throughout the growing season. The communities on soil surface and corn foliage were sampled during the V4, V8, and anthesis plant stages, and the subterranean community was sampled during the V2, V4, V8, and anthesis. Individual and grouped taxa included in the table represent those comprising ≥0.5% of the community abundance in their respective habitats and differed significantly between treatments. Collembola and mite abundances were excluded when determining commonly collected epigeic and subterranean taxa due to these species being a disproportionately large percentage of the total soil-dwelling community abundance. Results of repeated measures ANOVAs (α = 0.05) are presented for treatment effects with significant differences denoted with an asterisk.

**Table 2.** Predation on wax moth (*Galleria mellonella*) sentinels in corn monocultures and in corn possessing a mix of interseeded cover crops

Site year	**Wax moth sentinels eaten**		ANOVA
	Cover crops	Monoculture	
**Site 1**	31.3 ± 7.7	23.8 ± 9.7	*F*_1, 6_ = 0.37, *P* = 0.57
**Site 2**	35.0 ± 5.3	15.6 ± 5.0	*F*_1, 6_ = 7.01, *P* = 0.04*
**Across site years**	33.13 ± 4.37	19.69 ± 5.29	*F*_1, 31_ = 3.83, *P* = 0.07

Data represent mean ± SEM percent per plot consumed (*n* = 40 larvae per plot, 4 plots per site). Asterisks next to one-way ANOVA results represent significant treatment differences (α = 0.05).

**Table 3.** Invertebrate taxa observed actively eating sentinel wax moth larvae (*Galleria mellonella*) presented on the soil’s surface in corn

Class	Order	Family	*n*
**Insecta**	Orthoptera	Gryllidae	330
	Hymenoptera	Formicidae	119
	Coleoptera	Carabidae	31
		Coccinellidae	1
	Hemiptera	Geocoridae	2
**Arachnida**	Opiliones	Phalangiidae	37
	Araneae	Lycosidae	4
		Linyphiidae	1
**Gastropoda**	Stylommatophora	No I.D.	20
**Chilopoda**	Lithobiomorpha	No I.D.	1
**Diplopoda**	Julida	No I.D.	1
**Total**			547

In total, 640 sentinels were deployed and after 1 h; 211 larvae were actively being eaten by 1 or more invertebrates.

**Table 4.** Corn grain yields and plant densities from corn monocultures and cover crop-interseeded plots

Site year	Interseeded corn	Monoculture corn	ANOVA
**Yield (kg/Ha)**			
**Site 1**	1,481.61 ± 359.16	7,171.66 ± 381.94	*F*_1,7_ = 117.97, *P* < 0.01*
**Site 2**	13,416.37 ± 647.57	11,898.21 ± 250.61	*F*_1,7_ = 4.74, *P* = 0.07
**Site 3**	12,184.65 ± 284.19	12,483.99 ± 333.61	*F*_1,7_ = 0.45, *P* = 0.53
**Density (plants/Ha)**			
**Site 1**	68,897.04 ± 2,060.64	41,705.55 ± 2,866.98	*F*_1,7_ = 58.93, *P* < 0.01*
**Site 2**	71,674.42 ± 5,151.60	67,194.77 ± 1567.88	*F*_1,7_ = 0.17, *P* = 0.69
**Site 3**	72,794.33 ± 1,567.88	71,674.42 ± 895.93	*F*_1,7_ = 0.37, *P* = 0.56

Data presented are mean ± SEM values. Significant differences between treatments are indicated with an asterisk (α = 0.5).

## Supplementary Material

ieag096_Supplementary_Data

## References

[ieag096-B1] Abdin O , Coulman BE, Cloutier D, et al 1998. Yield and yield components of corn interseeded with cover crops. Agron. J. 90:63–68. 10.2134/agronj1998.00021962009000010012x

[ieag096-B2] Alarcón-Segura V , Grass I, Breustedt G, et al 2022. Strip intercropping of wheat and oilseed rape enhances biodiversity and biological pest control in a conventionally managed farm scenario. J. Appl. Ecol. 59:1513–1523. 10.1111/1365-2664.14161

[ieag096-B3] Altieri MA. 1999. The ecological role of biodiversity in agroecosystems. Invertebrate biodiversity as bioindicators of sustainable landscapes. Elsevier. p. 19–31.

[ieag096-B4] Altieri MA , Schmidt LL. 1986. Cover crops affect insect and spider populations in apple orchards. Calif. Agr. 40:15–17.

[ieag096-B5] Bennion LD , Ferguson JA, New LF, et al 2020. Community-level effects of herbicide-based restoration treatments: structural benefits but at what cost? Restoration Ecol. 28:553–563. 10.1111/rec.13118

[ieag096-B6] Bickerton MW , Hamilton GC. 2012. Effects of intercropping with flowering plants on predation of *Ostrinia nubilalis* (Lepidoptera: Crambidae) eggs by generalist predators in bell peppers. Environ. Entomol. 41:612–620. 10.1603/EN11249 22732620

[ieag096-B7] Bredeson MM , Lundgren JG. 2015. Thiamethoxam seed treatments have no impact on pest numbers or yield in cultivated sunflowers. J. Econ. Entomol. 108:2665–2671. 10.1093/jee/tov249 26340223

[ieag096-B8] Bredeson MM , Michels A, Welch KD, et al 2026. Invertebrate ­community structure in dominant agroecosystems of Saskatchewan, Manitoba, and North Dakota. Ann. Entomol. Soc. Am. 119:38–45. 10.1093/aesa/saaf037 41568079 PMC12815888

[ieag096-B9] Bröcher M , Ebeling A, Hertzog L, et al 2023. Effects of plant diversity on species-specific herbivory: patterns and mechanisms. Oecologia 201:1053–1066. 10.1007/s00442-023-05361-6 36964400 PMC10113292

[ieag096-B10] Coll M , Wajnberg E. 2017. Environmental pest management: a call to shift from a pest-centric to a system-centric approach. In: Coll M, Wajnberg E, editors. Environmental pest management: challenges for agronomists, ecologists, economists and policymakers. John Wiley and Sons Ltd. p. 1–18.

[ieag096-B11] CTIC 2017. Report of the 2016-17 National Cover Crop Survey. Joint publication of the Conservation Technology Information Center, the North Central Region Sustainable Agriculture Research and Education Program, and the American Seed Trade Association, West Lafayette, IN.

[ieag096-B12] Curran WS , Hoover RJ, Roth GW, et al 2018. Evaluation of cover crops drill-interseeded into corn across the Mid-Atlantic. Crops Soils 51:18–60. 10.2134/cs2018.51.0506

[ieag096-B13] DeFries RS , Foley JA, Asner GP. 2004. Land-use choices: balancing human needs and ecosystem function. Front. Ecol. Environ. 2:249–257. 10.1890/1540-9295(2004)002[0249:LCBHNA

[ieag096-B14] Fausti S , Kolady DE, Van der Sluis E, et al 2018. Extensive usage of insecticide and changing crop rotation patterns: a South Dakota case study. PLoS One. 13:e0208222. 10.1371/journal.pone.0208222 30496269 PMC6264870

[ieag096-B15] Fausti SW , McDonald TM, Lundgren JG, et al 2012. Insecticide use and crop selection in regions with high GM adoption rates. Renew. Agric. Food Syst. 27:295–304. 10.1017/S1742170511000561

[ieag096-B16] Fenster TLD , Oikawa PY, Lundgren JG. 2021. Regenerative almond production systems improve soil health, biodiversity, almond nutrition, and profit. Front. Sustain. Food Syst. 5:664359. 10.3389/fsufs.2021.664359

[ieag096-B17] Fiedler AK , Landis DA, Wratten SD. 2008. Maximizing ecosystem ­services from conservation biological control: the role of habitat management. Biol. Control. 45:254–271. 10.1016/j.biocontrol.2007.12.009

[ieag096-B18] Finke DL , Denno RF. 2002. Intraguild predation diminished in complex-structured vegetation: implications for prey suppression. Ecology 83:643–652. 10.2307/3071870

[ieag096-B19] Frank SD , Shrewsbury PM, Denno RF. 2010. Effects of alternative food on cannibalism and herbivore suppression by carabid larvae. Ecol. Entomol. 35:61–68. 10.1111/j.1365-2311.2009.01156.x

[ieag096-B20] Gurr GM , Wratten SD, Landis DA, et al 2017. Habitat management to suppress pest populations: progress and prospects. Annu. Rev. Entomol. 62:91–109. 10.1146/annurev-ento-031616-035050 27813664

[ieag096-B21] Horton DR , Jones VP, Unruh TR. 2009. Use of a new immunomarking method to assess movement by generalist predators between a cover crop and tree canopy in a pear orchard. Am. Entomol 55:49–56. 10.1093/ae/55.1.49

[ieag096-B22] Huber C , Zettl F, Hartung J, et al 2022. The impact of maize-bean intercropping on insect biodiversity. J. Appl. Ecol. 61:1–9. 10.1016/j.baae.2022.03.005

[ieag096-B23] Huss CP , Holmes KD, Blubaugh CK. 2022. Benefits and risks of intercropping for crop resilience and pest management. J. Econ. Entomol. 115:1350–1362. 10.1093/jee/toac045 35452091

[ieag096-B24] Hutchison WD , Burkness EC, Mitchell PD, et al 2010. Areawide suppression of european corn borer with *Bt* maize reaps savings to non-*Bt* maize growers. Science 330:222–225. 10.1126/science.1190242 20929774

[ieag096-B25] Jarvinen A , Hyvonen T, Raiskio S, et al 2023. Intercropping shifts the balance between generalist arthropd predators and oilseed pests towards natural pest control. Agric. Ecosyst. Environ. 348:108415. 10.1016/j.agee.2023.108415

[ieag096-B26] Jaworski CC , Thomine E, Rusch A, et al 2023. Crop diversification to promote arthropod pest management: a review. Agric. Communications 1:100004. 10.1016/j.agrcom.2023.100004

[ieag096-B27] Kirk DA , Martinez-Lanfranco JA, Forsyth DJ, et al 2025. Invertebrate diversity is shaped by farm management, edge effects and landscape context in the Prairie Pothole Region of Canada. Agric. Ecosyst. Environ. 377:109194. 10.1016/j.agee.2024.109194

[ieag096-B28] LaCanne C. 2017. Interactive effects of cover crops, invertebrate communities and soil health in corn production systems. Open Prairie Electronic Theses and Dissertations. p. 1195. https://openprairie.sdstate.edu/etd/1195/

[ieag096-B29] Landis DA , Menalled FD, Costamagna AC, et al 2005. Manipulating plant resources to enhance beneficial arthropods in agricultural landscapes. Weed Sci 53:902–908. 10.1614/WS-04-050R1.1

[ieag096-B30] Landis DA , Wratten SD, Gurr GM. 2000. Habitat management to conserve natural enemies of arthropod pests in agriculture. Annu. Rev. Entomol. 45:175–201. 10.1146/annurev.ento.45.1.175 10761575

[ieag096-B31] Landon AJ. 2008. The“how” of the three sisters: the origins of agriculture in Mesoamerica and the human niche. Online Dictionary of Invertebrate Zoology, UNL Digital Commons. https://digitalcommons.unl.edu/cgi/viewcontent.cgi?article=1039&context=nebanthro

[ieag096-B32] Langellotto GA , Denno RF. 2004. Responses of invertebrate natural enemies to complex-structured habitats: a meta-analytical synthesis. Oecologia 139:1–10. 10.1007/s00442-004-1497-3 14872336

[ieag096-B33] Letourneau DK , Armbrecht I, Rivera BS, et al 2011. Does plant diversity benefit agroecosystems? A synthetic review. Ecol. Appl. 21:9–21. 10.1890/09-2026.1 21516884

[ieag096-B34] Longcore T. 2003. Terrestrial arthropods as indicators of ecological restoration success in coastal sage scrub (California, U.S.A.). Restor. Ecol. 11:397–409. 10.1046/j.1526-100X.2003.rec0221.x

[ieag096-B35] Lundgren JG. 2009. Relationships of natural enemies and non-prey foods. Springer Science & Business Media. p. 23–43; 1–15.

[ieag096-B36] Lundgren JG , Fausti SW. 2015. Trading biodiversity for pest problems. Sci. Adv. 1:e1500558. 10.1126/sciadv.1500558 26601223 PMC4646789

[ieag096-B37] Lundgren JG , Fergen JK. 2010. The effects of a winter cover crop on *Diabrotica virgifera* (Coleoptera: Chrysomelidae) populations and beneficial arthropod communities in no-till maize. Environ. Entomol. 39:1816–1828. 10.1603/EN10041 22182547

[ieag096-B38] Lundgren JG , Fergen JK. 2011. Enhancing predation of a subterranean insect pest: a conservation benefit of winter vegetation in agroecosystems. Appl. Soil. Ecol. 51:9–16. 10.1016/j.apsoil.2011.08.005

[ieag096-B39] Lundgren JG , McDonald T, Rand TA, et al 2015. Spatial and numerical relationships of arthropod communities associated with key pests of maize. J. Appl. Entomol. 139:446–456. 10.1111/jen.12215

[ieag096-B40] Lundgren JG , Shaw JT, Zaborski ER, et al 2006. The influence of organic transition systems on beneficial ground-dwelling arthropods and predation of insects and weed seeds. Renew. Agric. Food Syst. 21:227–237. 10.1079/RAF2006152

[ieag096-B41] Lundgren JG , Welch KD, Bredeson MM, et al 2026. Regenerative agriculture: connecting soil carbon storage, biodiversity, and profit. Environ. Res: Food Syst. 3:035020. 10.1088/2976-601X/ae8f4e

[ieag096-B42] Manandhar R , Wright MG. 2016. Effects of interplanting flowering plants on the biological control of corn earworm (Lepidoptera: Noctuidae) and thrips (Thysanoptera: Thripidae) in sweet corn. J. Econ. Entomol. 109:113–119. 10.1093/jee/tov306 26500338

[ieag096-B43] Meehan TD , Werling BP, Landis DA, et al 2012. Pest-suppression potential of Midwestern landscapes under contrasting bioenergy scenarios. PLoS One. 7:e41728. 10.1371/journal.pone.0041728 22848582 PMC3405014

[ieag096-B44] Meng L , Zhang A, Wang F, et al 2015. Arbuscular mycorrhizal fungi and rhizobium facilitate nitrogen uptake and transfer in soybean/maize intercropping system. Front. Plant Sci. 6:339. 10.3389/fpls.2015.00339 26029236 PMC4429567

[ieag096-B45] Mir MS , Saxena A, Kanth RH, et al 2022. Role of intercropping in sustainable insect-pest management: a review. IJECC. 12:3390–3404. 10.9734/ijecc/2022/v12i111390

[ieag096-B46] Moreira X , Abdala-Roberts L, Rasmann S, et al 2016. Plant diversity effects on insect herbivores and their natural enemies: current thinking, recent findings, and future directions. Curr. Opin. Insect Sci. 14:1–7. 10.1016/j.cois.2015.10.003 27436639

[ieag096-B47] Mourtzinis S , Krupke CH, Esker PD, et al 2019. Neonicotinoid seed treatments of soybean provide negligible benefits to US farmers. Sci. Rep. 9:11207. 10.1038/s41598-019-47442-8 31501463 PMC6733863

[ieag096-B49] Nemec KT , Allen CR, Danielson SD, et al 2014. Responses of predatory invertebrates to seeding density and plant species richness in experimental tallgrass prairie restorations. Agric. Ecosyst. Environ 183:11–20. 10.1016/j.agee.2013.10.024

[ieag096-B50] Nomikou M , Sabelis MW, Janssen A. 2010. Pollen subsidies promote whitefly control through the numerical response of predatory mites. BioControl 55:253–260. 10.1007/s10526-009-9233-x

[ieag096-B51] Ojala R , Huhta V. 2001. Dispersal of microarthropods in forest soil. Pedobiologia. (Jena) 45:443–450. 10.1078/0031-4056-00098

[ieag096-B52] Orr DB , Landis DA, Mutch DR, et al 1997. Ground cover influence on microclimate and *Trichogramma* (Hymenoptera: Trichogrammatidae) augmentation in seed corn production. Environ. Entomol. 26:433–438. 10.1093/ee/26.2.433

[ieag096-B53] Pearsons K , Tooker J. 2017. In-field habitat management to optimize pest control of novel soil communities in agroecosystems. Insects 8:82. 10.3390/insects8030082 28783074 PMC5620702

[ieag096-B54] Pecenka D , Welch KD, Boe A, et al 2026. Regenerative wheat management: the relative effects of farming practices on the biodiversity of invertebrates. Insect Conserv. Divers. 2026:1–15. 10.1111/icad.70105

[ieag096-B55] Pierre JF , Latournerie-Moreno L, Garruña R, et al 2022. Effect of maize-legume intercropping on maize physio-agronomic parameters and beneficial insect abundance. Sustainability 14:12385. 10.3390/su141912385

[ieag096-B56] Rashford BS , Walker JA, Bastian CT. 2011. Economics of grassland conversion to cropland in the prairie pothole region. Conserv. Biol. 25:276–284. 10.1111/j.1523-1739.2010.01618.x 21166716

[ieag096-B57] Rerkasem K , Rerkasem B. 1988. Yields and nitrogen nutrition of intercropped maize and ricebean (*Vigna umbellata* [Thumb.] Ohwi and Ohashi). Plant Soil 108:151–162. 10.1007/BF02370110

[ieag096-B58] Rinehart L. 2006. Switchgrass as a bioenergy crop. National Center for Appropriate Technology. http://attra. ncat. org/attra-pub/PDF/switchgrass. pdf

[ieag096-B59] Samu F , Szinetar C. 2002. On the nature of agrobiont spiders. J. Arachnol. 30:389–402. 10.1636/0161-8202(2002)030[0389:OTNOAS

[ieag096-B60] Schmid RB , Lehman RM, Brözel VS, et al 2015. Gut bacterial symbiont diversity within beneficial insects linked to reductions in local bio­diversity. Ann. Entomol. Soc. Am. 108:993–999. 10.1093/aesa/sav081

[ieag096-B61] SDSU. 2019. South Dakota State University pest management guide-corn. https://extension.sdstate.edu/2019-south-dakota-pest-management-guides

[ieag096-B62] Siemann E , Haarstad J, Tilman D. 1999. Dynamics of plant and arthropod diversity during old field succession. Ecography 22:406–414. 10.1111/j.1600-0587.1999.tb00577.x

[ieag096-B63] Slosser JE , Parajulee MN, Bordovsky DG. 2000. Evaluation of food sprays and relay strip crops for enhancing biological control of bollworms and cotton aphids in cotton. Internat. J. Pest. Manage. 46:267–275. 10.1080/09670870050206037

[ieag096-B64] Standen V. 2000. The adequacy of collecting techniques for estimating species richness of grassland invertebrates. J. Appl. Ecol. 37:884–893. 10.1046/j.1365-2664.2000.00532.x

[ieag096-B65] Stevenson K , Anderson RV, Vigue G. 2002. The density and diversity of soil invertebrates in conventional and pesticide free corn. Trans. Illinois State Acad. Sci. 95:1–9.

[ieag096-B66] Suding KN , Gross KL. 2006. The dynamic nature of ecological systems: multiple states and restoration trajectories. In: Falk DA, Palmer MA, Zedler JB, editors. Foundations in restoration ecology. Princeton University Press. p. 190–209.

[ieag096-B67] USDA-ERS. 2026. United States Department of Agriculture Economic Research Service. Total land use. (Accessed 29 September 2026). https://www.ers.usda.gov/topics/farm-economy/land-use-land-value-tenure/major-land-uses

[ieag096-B68] USDA-NASS. 2026. United States Department of Agriculture National Agricultural Statistics Service. Statistics by subject: corn. (Accessed 29 September 2026). https://www.nass.usda.gov/

[ieag096-B69] Venzon M , Rosado MC, Euzébio DE, et al 2006. Suitability of leguminous cover crop pollens as food source for the green lacewing *Chrysoperla externa* (Hagen) (Neuroptera: Chrysopidae). Neotrop. Entomol. 35:371–376. 10.1590/s1519-566x2006000300012 18575698

[ieag096-B70] Wallander S , Smith D, Bowman M, et al 2022. Cover crop trends, programs, and practices in the United States. USDA-ERS, Economic Information Bulletin Number 222. p. 33. https://www.ers.usda.gov/publications/100550

[ieag096-B71] Welch KD , Lundgren JG. 2016. An exposure-based, ecologically driven framework for selection of indicator species for insecticide risk assessment. Food Webs 9:46–54. 10.1016/j.fooweb.2016.02.004

[ieag096-B72] Wiedenmann RNW , Smith JW. Jr. 1997. Attributes of natural enemies in ephemeral crop habitats. Biol. Control. 10:16–22. 10.1006/bcon.1997.0544

[ieag096-B73] Wimberly MC , Janssen LL, Hennessy DA, et al 2017. Cropland expansion and grassland loss in the eastern Dakotas: new insights from a farm-level survey. Land Use Policy. 63:160–173. 10.1016/j.landusepol.2017.01.026

[ieag096-B74] Yu Y , Stomph T-J, Makowski D, et al 2015. Temporal niche differentiation increases the land equivalent ratio of annual intercrops: a meta-analysis. Field Crops Res. 184:133–144. 10.1016/j.fcr.2015.09.010

