# Supplementary material for: Interseeding cover crops into established corn fields affects invertebrate abundance and diversity and elevates the activity of generalist predators
Source: J Insect Sci. 2026 Sep 16;26(5):ieag096. doi: 10.1093/jisesa/ieag096 (PMC13581462; doi:10.1093/jisesa/ieag096)

Figure S1. Planter designed to interseed cover crops between corn rows. One pair of double-disc openers planted a single row of cover crops equidistantly between corn rows spaced 76 cm. The planter is capable of planting seven rows in a single pass.

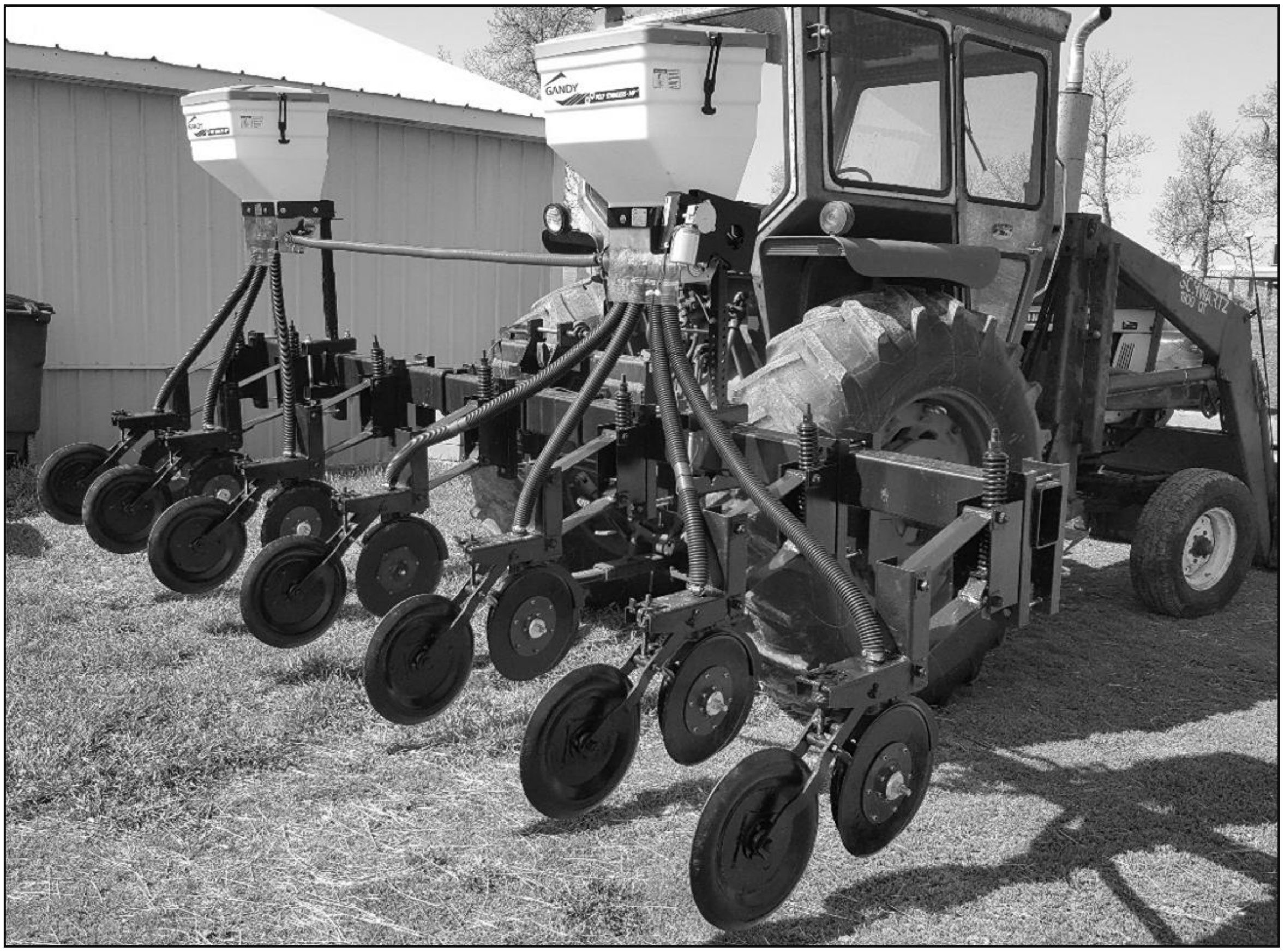

Supplement: Supplementary file 1 [file ieag096_supplementary_data.pdf]
